# COVID-19 response and mitigation: a call for action

**DOI:** 10.2471/BLT.20.285322

**Published:** 2021-02-01

**Authors:** Viroj Tangcharoensathien, Poonam Singh, Anne Mills

**Affiliations:** a International Health Policy Program, Ministry of Public Health, Tiwanon Road, Muang District, Nonthaburi 11000, Thailand.; b World Health Organization, Regional Office for South-East Asia, New Delhi, India.; c London School of Hygiene & Tropical Medicine, University of London, London, England.

At the end of 2020, 77 million people had tested positive for the severe acute respiratory syndrome coronavirus 2 and of these, 1.7 million had died from coronavirus disease 2019 (COVID-19). Of the total 23.9 million active cases on 10 January 2021, 108 368 (0.5%) were critically ill,^1^ requiring significant health-care resources. Of the COVID-19 survivors who have lasting sequelae, many will require continuing treatment and hence will need long-term health care. A systematic review covering 166 countries reported 152 888 infections and 1413 deaths among health-care workers as of 8 May 2020.^2^ The significant temporary reduction of health-care worker capacity through periods of infection and isolation, as well as permanent attrition through death or severe long-term sequalae, hamper public health responses and clinical management. 

Before the COVID-19 pandemic, health systems in most low- and middle-income countries were stretched, contributing to a lack of universal access to health services. During the pandemic, many governments have redirected resources to the COVID-19 response, further stretching the health system and disrupting other disease prevention and treatment services. This issue of the *Bulletin* goes beyond the health sector to examine some of the wider issues of governance as they affect pandemic response and the introduction of diverse measures at different times.^3^ Effective and decisive whole-of-government responses^4^ and female leadership have been associated with better health outcomes.^5^ Other good governance attributes such as transparency, effective public communication by the highest level of government and accountability of decision-makers have also been shown to contribute to better containment.^6^

In the United States of America (USA), analysis of risk communications shows inconsistent messaging on transmission risks, the need to wear masks and stay at home, and the use of disinfectants and hand sanitizer.^7^

Trust between governments and their constituencies has contributed to effective containment,^8^ particularly reciprocal trust – both horizontally among people and vertically between people and their governments.^9^ A study found that trust in government communications was associated with a higher perceived threat of infection and greater likelihood of practicing protective measures among Singaporeans.^10^

Evidence is accumulating that some of the risk factors associated with COVID-19 infection and severity, such as air pollution in the form of fine particulate matter (PM_2.5_) and tobacco use, are affected by policies across government sectors. Most countries have national air quality standards that are less stringent than the WHO-established annual limit of < 10 µg PM_2.5 _per cubic metre.^11^ Exposure to air pollution, including PM_2.5_ and nitrogen dioxide, increase the expression of the angiotensin-converting enzyme 2 on epithelial cell surfaces of the respiratory tract, and this overexpression is positively correlated with COVID-19 severity.^12^ Furthermore, smoking among young people in the USA is a significant risk factor for COVID-19 infection.^13^

The global response to the pandemic has been inadequate. Wealthier countries will naturally put their own people first, and poorer countries have little political or economic leverage to change this stance; however, donor governments could help low- and middle-income countries deal with the pandemic. The unhealthy geopolitics that hamper global responses and how international instruments that address climate change can be applied to pandemics, are also discussed in this theme issue.^14^^,^^15^ Ways to address the geopolitical status quo, through understanding of the social, commercial and ecosocial determinants of health, are suggested.^16^

Financial rescue packages need to address fundamental economic and social challenges revealed by the pandemic, particularly the high level of informality in the workforce, weak social safety nets and inability to access care, which disproportionately affect vulnerable populations. Estimations show that most economic loss from COVID-19 in China is productivity loss, notably from travel restriction measures, since domestic and international travel accounts for 382.3 billion United States dollars (US$) or 2.7% of China’s gross domestic product of US$ 14.14 trillion.^17^ The ethical implications of using immunity certificates to facilitate travel and resume other economic activities are discussed by Voo et al.^18^

This issue will be virtually launched at the 2021 Prince Mahidol Award Conference under the theme *COVID-19: advancing towards an equitable and healthy world*. 
